# Genome-Wide Identification and Transcriptional Analysis of Arabidopsis *DUF506* Gene Family

**DOI:** 10.3390/ijms222111442

**Published:** 2021-10-23

**Authors:** Sheng Ying

**Affiliations:** Noble Research Institute LLC, Ardmore, OK 73401, USA; yingshen@msu.edu

**Keywords:** abiotic stress, arabidopsis, domain of unknown function (DUF), domain-structure, evolutionary analysis, expression profiling

## Abstract

The Domain of unknown function 506 (*DUF506*) family, which belongs to the PD-(D/E)XK nuclease superfamily, has not been functionally characterized. In this study, 266 *DUF506* domain-containing genes were identified from algae, mosses, and land plants showing their wide occurrence in photosynthetic organisms. Bioinformatics analysis identified 211 high-confidence *DUF506* genes across 17 representative land plant species. Phylogenetic modeling classified three groups of plant *DUF506* genes that suggested functional preservation among the groups based on conserved gene structure and motifs. Gene duplication and Ka/Ks evolutionary rates revealed that *DUF506* genes are under purifying positive selection pressure. Subcellular protein localization analysis revealed that *DUF506* proteins were present in different organelles. Transcript analyses showed that 13 of the Arabidopsis *DUF506* genes are ubiquitously expressed in various tissues and respond to different abiotic stresses and ABA treatment. Protein-protein interaction network analysis using the STRING-DB, AtPIN (*Arabidopsis thaliana* Protein Interaction Network), and AI-1 (Arabidopsis Interactome-1) tools indicated that AtDUF506s potentially interact with iron-deficiency response proteins, salt-inducible transcription factors, or calcium sensors (calmodulins), implying that *DUF506* genes have distinct biological functions including responses to environmental stimuli, nutrient-deficiencies, and participate in Ca(2+) signaling. Current results provide insightful information regarding the molecular features of the *DUF506* family in plants, to support further functional characterizations.

## 1. Introduction

Domain of unknown function (DUF) is a conserved protein domain that has not been extensively characterized [1]. Designation as a DUF domain was derived from two bacterial motifs (DUF1 and 2), which were, respectively, renamed as GGDEF and EAL domains, based on their featured peptides [1,2]. The availability of whole genome sequence information has enabled the generation of a list of DUF-classified proteins in a wide range of organisms with a considerable portion (31%, 5889 DUFs) found in the 19,179 entries in the Pfam database (34.0, https://pfam.xfam.org/, accessed on August 2021) [3]. Numerous *DUF* genes have been studied in different species of plants. In Arabidopsis, *DUF6* [4], *DUF26* [5], *DUF538* [6], *DUF579* [7], *DUF581* [8], *DUF617/MIZ* [9], *DUF642* [10], *DUF647* [11], *DUF724* [12], *DUF784* [13], *DUF827* [14], *DUF1117* [15] and *DUF4228* [16] have been functionally characterized, and these studies have revealed the involvement of DUF-domain containing proteins in diverse biological processes. For example, *At5g06700/TRICHOME BIREFRINGENCE/TBR* and its homolog *At5g01360/TRICHOME BIREFRINGENCE-LIKE 3/TBL3*, which belong to the plant-specific DUF231 family, function in maintaining homeostasis of secondary wall cellulose synthesis in Arabidopsis [17]. Furthermore, another DUF231 member, At3g55990/ESK1/TBL29, was shown to be a negative regulator of freeze tolerance [18]. A recent evolutionary study revealed that *DUF231* genes are found in all land plants and conduct conserved functions [19].

The *DUF506* family belongs to the PD-(D/E)XK nuclease superfamily [20], which exists in all organisms. Most of the studies of *DUF506* domain-containing proteins involve biochemical, mutagenesis, and structural biological approaches [21,22]. Currently, this superfamily includes approximately 22,900 proteins classified into 121 family branches [23]. However, due to low protein sequence similarity, *DUF506* genes display extremely distant homologs among families [20] making it difficult to elucidate the molecular and biological functions of individual *DUF506* genes. Recently, a phosphate-stress response *DUF506* gene (*At3g25240/REPRESSOR OF EXCESSIVE ROOT HAIR GROWTH 1/RXR1*) in Arabidopsis was experimentally demonstrated to repress root hair elongation via interaction with RAB GTPase regardless of P supplied status [24]. Furthermore, overexpression of the *RXR1* homolog (*Bradi2g58590*) in the monocot model plant, Brachypodium, exhibited the identical short root hair phenotype, suggesting the conservative function of *RXR1* in regulation of plant cell development. Thus, the aim of this work was to conduct an extensive bioinformatics, expression profiling and subcellular analyses of plant *DUF506* genes, to uncover their additional biological functions.

In this study, 211 *DUF506* genes from 17 land plant species were identified with high confidence. These *DUF506* genes were subjected to evolutionary analysis. In addition, subcellular localization, expression profiling analysis, and putative interactor(s) predictions of *DUF506* family members not only extend the current knowledge of the PD-(D/E)XK superfamily in plants, but also provide beneficial insights for further studies into the distinct functions, such as abiotic stress responses, or regulation of calcium signaling.

## 2. Results

### 2.1. Genome-Wide Survey and Phylogenetic Clustering of DUF506 Genes in Plants

**Figure 1:**
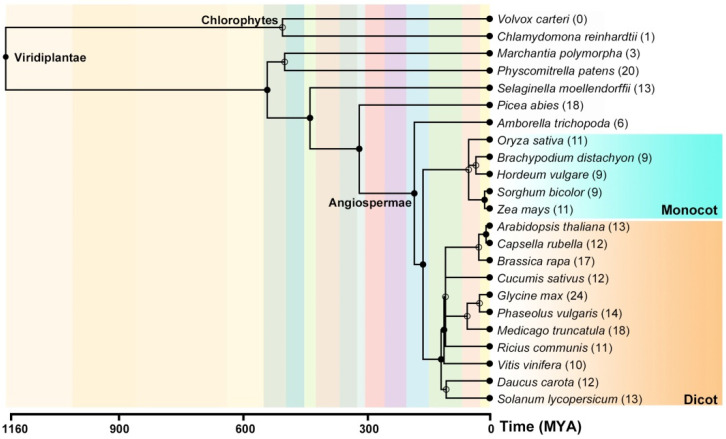
Phylogeny distribution of *DUF506* genes across 23 species used in this study. The number of high confidence *DUF506* genes is listed in parentheses. The order of tree branches and divergence time are derived from the TimeTree database (http://www.timetree.org/, accessed on August 2021). Monocot species are highlighted in the rectangle of teal color, whereas the dicot species are masked by rectangle in yellow color.

To extend current understanding of the *DUF506* gene family, 323 putative *DUF506* candidate gene sequences were extracted from the NCBI and Phytozome databases. Gene sequences encoding *DUF506* domain-containing proteins were identified from 23 representative species, including Chlorophyta, Bryophyta, Lycophyta, Gymnospermae and Angiospermae (basal angiosperm, monocots and eudicots). Then, comprehensive protein sequences alignment [25] and domain alignment (HMM, Hidden Markov Model) [26] were respectively conducted to eliminate candidates with low integrity or incomplete domains. The remaining 266 *DUF506* genes were then subjected to evolutionary analysis (Figure 1). Notably, no *DUF506* genes were identified from *Volvox carteri*, and only one candidate was discovered from another chlorophyte (*Chlamydomona reinhardtii*). Generally, the dicot species have more *DUF506* genes compared to monocots. The number of *DUF506* gene sequences was highest in soybean (24), followed by two other legumes.

**Figure 2:**
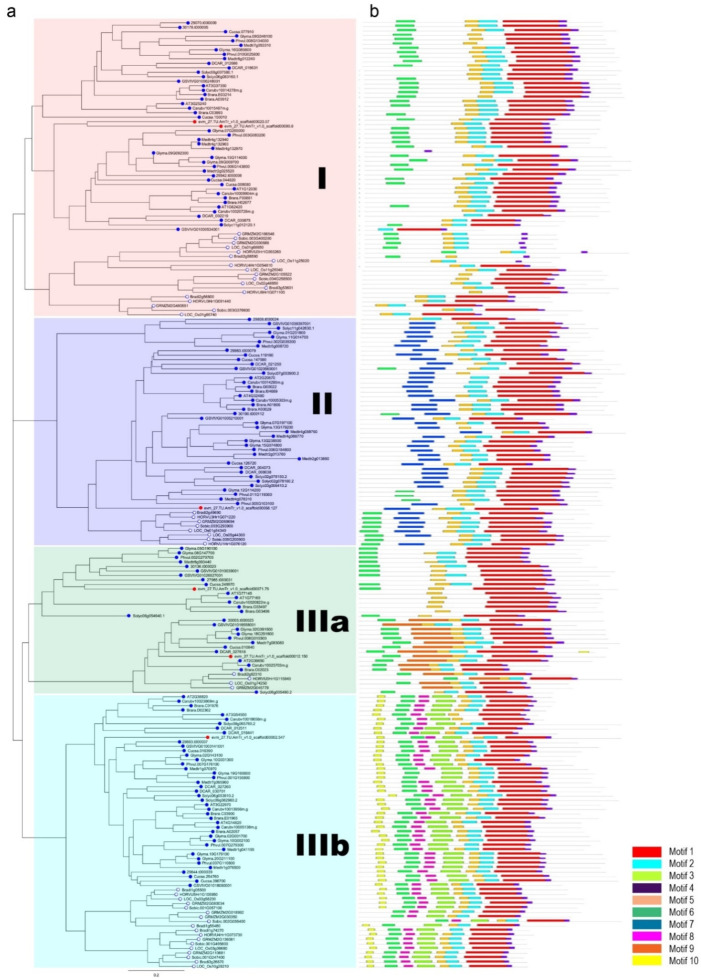

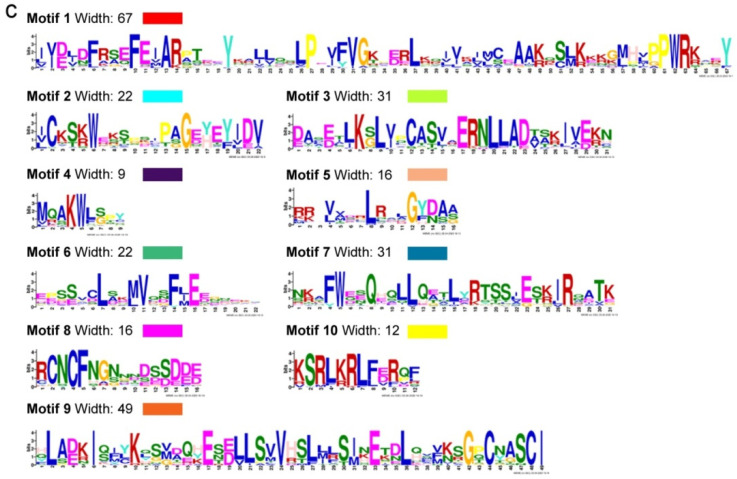
Phylogenetic representation and distribution of the conserved motifs in *DUF506* among angiosperms. (**a**) The phylogenetic tree was constructed based on full-length *DUF506* amino acid sequence alignments from 17 species. *DUF506* from monocots are represented by blue hollow dots, while those from dicots are represented by blue solid dots. *DUF506* from *Amborella trichopoda* (basal angiosperm) are represented by red solid dots. The distribution of *DUF506* conserved motifs (**b**) and the corresponding sequence logos (**c**) in plants are shown. Note that, the conserved β-sheet structures are mainly localized in Motif 2.

A phylogenetic tree was constructed to examine the evolutionary relationships among the 211 *DUF506* candidates derived from 17 species in Angiosperms, using full-length protein sequence alignments. These *DUF506* proteins were classified into three distinct groups (Ⅰ, Ⅱ, and Ⅲ), consisting of 66, 51, and 94 members, respectively (Figure 2a, Table S1). Group Ⅲ was further divided into two subgroups, named Ⅲa and Ⅲb, with 33 and 61 members, respectively. *DUF506* proteins were found to be evenly distributed in each group (Table S2). For example, in Arabidopsis, there were four, two and seven *DUF506* members in group Ⅰ, group Ⅱ, and group Ⅲ, respectively. The lengths of Arabidopsis *DUF506* protein ranged from 261 to 371 amino acid residues, while their predicted theoretical pI (Isoelectric point, the pH value at which the total charge on the protein is zero) varied from 5.02 to 8.96 (Table 1). Importantly, monocot and eudicot *DUF506* proteins clustered into different branches (Figure 2a). These results indicate that classification of *DUF506* members is largely concordant with the species tree (Figure 1).

### 2.2. Motif Conservation within Diverse DUF506 Family Groups

Within the *DUF506* family, ten putative conserved motifs between five and one hundred amino acid residues were predicted using MEME [27]. The corresponding motif sequence logos and their distribution along the sequence are illustrated in Figure 2b,c. Generally, group Ⅲa *DUF506* members had the highest motif abundance. The most ubiquitous was motif 4 and was present in all (211/211) *DUF506* proteins, while motif 1 (204/211), motif 2 (184/211), and motif 5 (203/211) were present in most *DUF506* proteins. In contrast, motifs 7 and 9 were exclusively found in group Ⅰ and Ⅲa, respectively. Motifs 3, 6, 8, and 10 were present only in group Ⅲb. Overall, *DUF506* members from the same groups generally share distinctive motif compositions, indicating that functional similarities within groups and the diversification among groups.

### 2.3. Characterization of Chromosomal Distribution, Gene Synteny, and Sequence Structure of Arabidopsis DUF506 Genes

The localization of *DUF506* genes on Arabidopsis chromosomes was visualized through ePlant website (https://bar.utoronto.ca/eplant/, accessed on May 2021). The thirteen Arabidopsis *DUF506* genes were found on chromosomes 1–4 (Figure S1).

Gene synteny analysis revealed three segmental duplication events involving six Arabidopsis *DUF506* genes (Figure S1, Table 2), whereas no tandem duplication was found. These results implied that the Arabidopsis *DUF506* gene family expanded during evolution through gene segmental duplication. The ratio of nonsynonymous nucleotide substitutions (Ka), which led to amino acid altering, versus synonymous nucleotide substitutions (Ks), which did not change the amino acid sequence, was used to determine the selective pressure during genome evolution after gene duplication events [28]. Ka/Ks ratios for all three duplicated gene pairs are below 1, indicating that gene functional conservation during evolution, and suggesting that the Arabidopsis *DUF506* gene family underwent purifying selection after the duplication events. Purifying selection of *DUF506* genes was also found in monocot species, such as Brachypodium, rice, maize and sorghum (Table S4).

**Figure 3:**
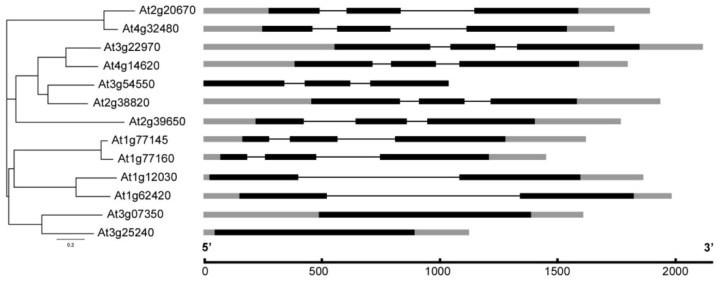
Phylogenetic analysis and exon-intron distribution of Arabidopsis *DUF506* genes. Exon, intron and UTR are represented by black rectangle boxes, black lines and grey rectangle boxes, respectively.

In addition, exon-intron distributions were examined to determine structural diversity among *DUF506* genes by using GSDS 2.0. The results showed almost 58% (122/211) of *DUF506* genes contained more than two exons (Figure S2, and Table S3). For example, nine of the Arabidopsis *DUF506* genes included three exons (Figure 3), and three duplicated gene pairs kept the identical exon-intron structure.

### 2.4. Wide Spread Localization of Arabidopsis DUF506 Proteins

**Figure 4:**
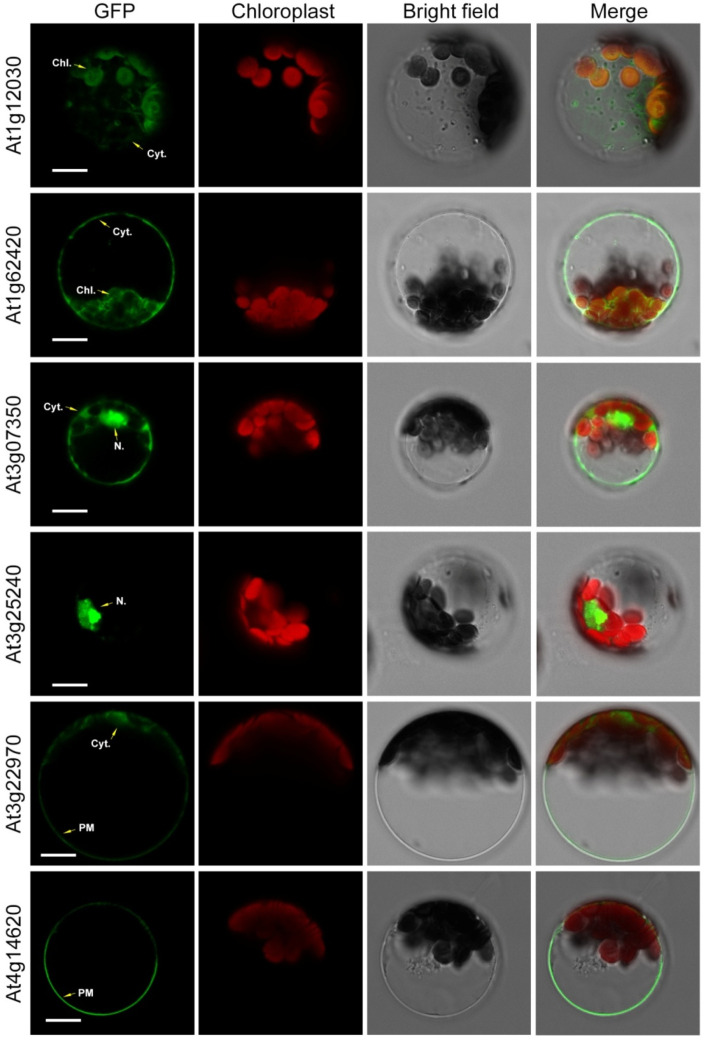
Subcellular localization of Arabidopsis *DUF506* proteins visualized by transient expression of GFP fusion constructs in Arabidopsis mesophyll protoplasts. Chl., chloroplast; Cyt., cytoplasm; N., nucleus; PM, plasma membrane. Scale bar, 10 µm.

To study the subcellular localization of *DUF506* proteins in Arabidopsis, six representative Arabidopsis *DUF506* genes were cloned and transferred into the pEarleyGate 103 plasmid [29] to generate AtDUF506-GFP fused proteins driven by the CaMV35S promoter. Constructs were individually introduced into Arabidopsis mesophyll protoplasts using polyethylene glycol (PEG)-mediated transfection [30]. The resulting transient expression patterns are shown in Figure 4. The duplicated gene pairs were consistently expressed in identical organelles. For instance, At1g12030 and At1g62420 were localized in both the chloroplast and cytoplasm, while At3g22970 and At4g14620 were mainly in the plasma membrane, and slightly in the cytoplasm. Conversely, At3g25240 was concentrated in the nucleus and At3g07350 was present in the nucleus and cytoplasm. These results suggest that *DUF506* proteins play diverse physiological roles based on their distinct patterns of organelle localization.

### 2.5. Tissue Expression Profiles of Arabidopsis DUF506 Genes

**Figure 5:**
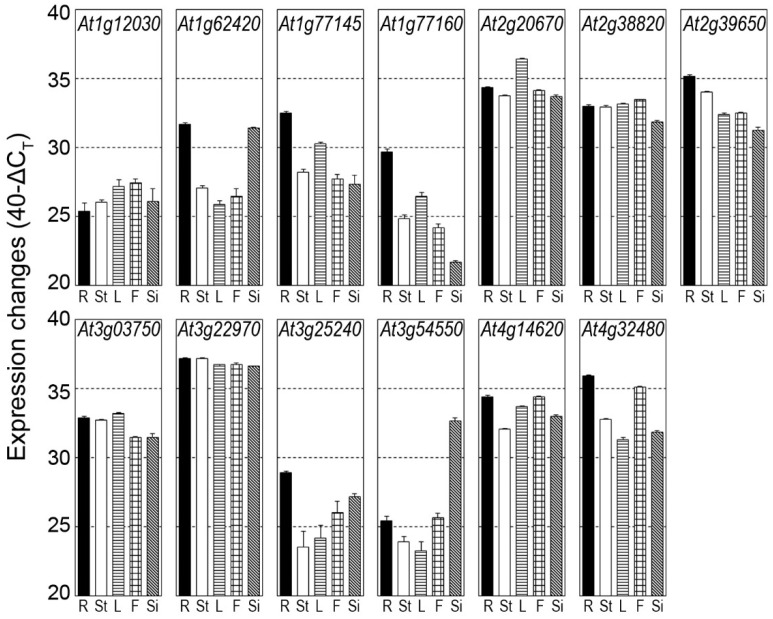
Expression profile of Arabidopsis *DUF506* genes in different tissues. R, root; St, stem; L, leave; F, flower; Si, silique. Changes of gene expression level between tissues are interpreted as described in [31]. Expression levels are given on a log scale expressed as 40^−ΔCT^, where ΔCT is the difference in qRT-PCR threshold cycle number between the respective gene and the reference gene (*GAPDH*); 40 therefore equals the expression level of *GAPDH*; the number 40 was chosen because the PCR run stops after 40 cycles. The fold difference in expression is 2^ΔΔCT^ when PCR efficiency is 2 (e.g., an ordinate value of 34 represents 16-fold lower expression than a value of 38).

**Figure 6:**
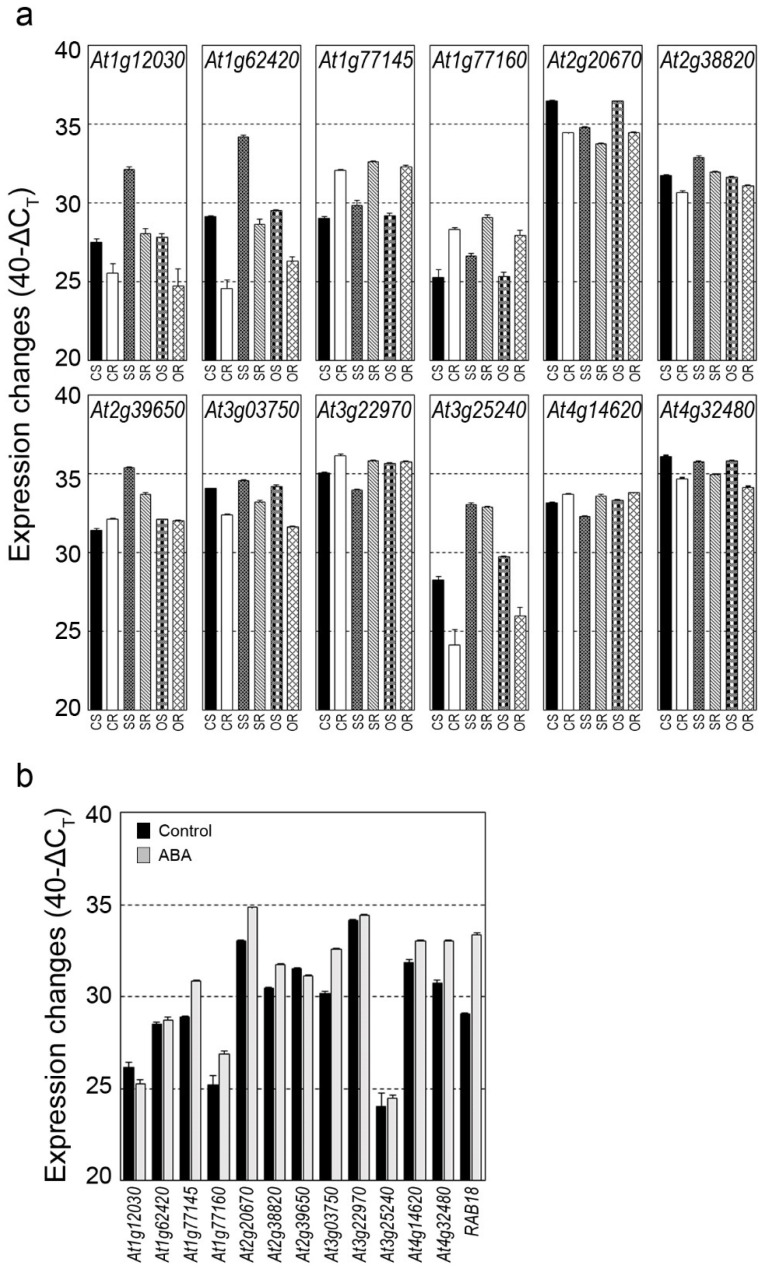
Expression profile of Arabidopsis *DUF506* genes in response to salt or osmotic stress (**a**) and Abscisic acid (ABA) treatment (**b**). CS, control shoot tissue; CR, control root tissue; SS, salt stress shoot tissue; SR, salt stress root tissue; OS, osmotic stress (mannitol) shoot tissue; OR, osmotic stress root tissue. *At5g66400/RAB18*, which is strongly induced by ABA, was used as positive external control (**b**).

To characterize the expression patterns of the Arabidopsis *DUF506* gene family, their transcript levels were examined in root, rosette leaves, stems, flowers, and siliques, using quantitative, real-time PCR (qRT-PCR). Arabidopsis *DUF506* gene transcripts were detected in all tested tissues and exhibited different expression patterns (Figure 5). The *At3g22970* transcript was abundant in all examined tissues, whereas the *At3g25240* transcript had a comparably low abundance. A few *DUF506* transcripts had much higher abundance in specific tissues or organs. For example, the *At3g54550* transcript was at least 50 times more abundant in siliques than in flowers or roots. Interestingly, in adult plants the *At1g62420* transcript was much more abundant in roots than in leaves, while in young seedlings, abundance was much higher in shoots than roots (Figure 6a). Such results suggest that *DUF506* genes might perform different functions during development of Arabidopsis plants.

### 2.6. Response of Arabidopsis DUF506 Gene Transcripts to Abiotic Stress and Abscisic Acid Treatments

Expression changes of Arabidopsis *DUF506* genes in young seedlings, under salt, osmotic, and Abscisic acid (ABA) treatment were investigated (Figure 6a). Due to the low expression level in seedlings, the *At3g54550* transcript was undetectable under all conditions. Under salt stress, *At1g12030*, *At1g62420*, *At1g77160*, *At2g38820*, *At2g39650*, and *At3g25240* were highly induced in shoot and root tissues (fold change [FC] ≥ 2), whereas *At1g77145* and *At3g07350* were slightly up-regulated (1 ≤ FC ≤ 2). By contrast, *At2g20670*, *At3g22970* and *At4g14620* were significantly down-regulated in shoots (FC ≥ 2). Except for *At1g62420* and *At3g25240*, no transcript changes were observed during osmotic stress.

Abscisic acid (ABA) has been shown to play an important role in stress responses and tolerance to abiotic stresses [32]. Most of the salt stress responsive Arabidopsis *DUF506* genes, including *At1g77145*, *At1g77160*, *At2g20670*, *At2g38820*, *At3g07350*, and *At4g14620*, positively responded to ABA treatment, while *At1g62420*, *At2g39650*, and *At3g25240* did not (Figure 6b). Meanwhile, expression of Arabidopsis *DUF506* genes were explored under heat and cold stress available through online public transcriptome databases (GENEVESTIGATOR^®^ and EMBL-EBI/ArrayExpress). *At3g25240* and *At1g62420* were highly induced, but *At2g20670* and *At3g07350* were significantly repressed by both temperature stress conditions (FC ≥ 2).

Stress-related *cis*-elements in the promoter regions (2 kb upstream of start codon) of the Arabidopsis *DUF506* genes were also examined and identified (Figure S3). At least two copies of ABRE (ABA-responsive element) were found upstream of Arabidopsis *DUF506* genes with the exception of *At2g20670*. The transcripts changes of the majority of Arabidopsis *DUF506* genes (8/13, Figure 6b) under ABA treatment suggest that some of them might be involved in integrating ABA-related signaling pathways with abiotic stress responses.

### 2.7. Putative Interactors of Arabidopsis DUF506 Proteins

**Figure 7:**
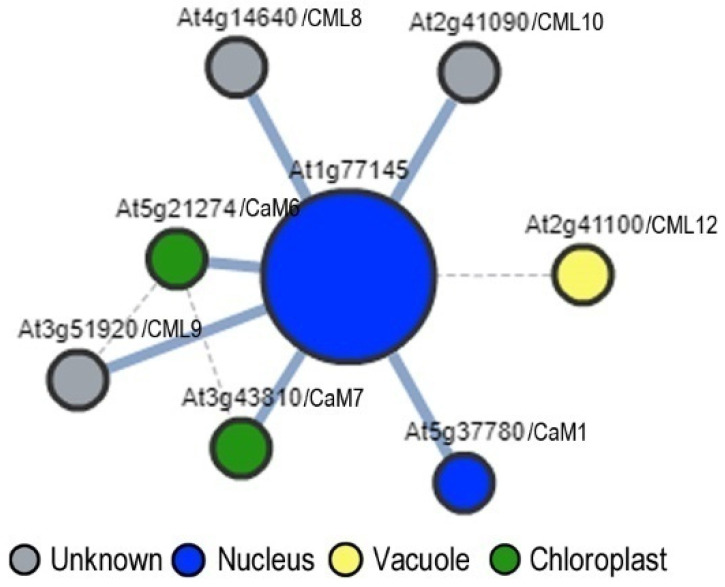
Predicted protein-protein interactions of At1g77145 and calmodulin/-like (CaM/CML). The interaction was predicted using AI-1 and the graph was generated by Arabidopsis interactions viewer (https://bar.utoronto.ca/interactions/cgi-bin/arabidopsis_interactions_viewer.cgi, accessed on June 2021). The color of nodes represents the subcellular localization predicted by SUBA (http://suba.live/, accessed on June 2021), and the thickness of lines represents the confident values of interactions. At2g41090, calmodulin like 10/CML10; At2g41100, calmodulin like 12/CML12; At3g43810, calmodulin 7/CaM7; At3g51920, calmodulin like 9/CML9; At4g14640, calmodulin like 8/CML8; At5g21274, calmodulin 6/CaM6; At5g37780, calmodulin 1/CaM1.

To further uncover potential biological functions of *DUF506* in Arabidopsis, protein-protein interaction analysis was conducted using three different online search tools (Table S6). STRING-DB [33] (v11.0, https://string-db.org/, accessed on May 2021) identified seventy-five potential interactors for nine Arabidopsis *DUF506* proteins, mainly based on coexpression profiles and text mining. Notably, several iron-deficiency related proteins (e.g., At1g47400/IRONMAM1, At5g67370/CGLD27, and At5g03570/IREG2) interacted with At1g12030, suggested its regulatory role in Fe signaling. Moreover, At2g39650 exhibited tight relationships with several salt stress regulators, such as salt-inducible ZINC Finger transcription factors (e.g., At3g55980/SZF1, At2g40140/SZF2, and At1g27730/ZAT10). On the other hand, the AtPIN and AI-1 programs only predicted two interactors of Arabidopsis *DUF506* proteins. Remarkably, At1g77145 was predicted and has been experimentally proven to extensively interact with different calmodulins (CaMs, Figure 7, [34]), which are small calcium-binding proteins that function as second messengers in plant Ca^2+^-signaling [35]. The CaMs were identified from both databases, suggesting that At1g77145 might participate in Ca^2+^/CaM mediating signal transduction.

## 3. Discussion

Although the genome draft of Arabidopsis was released over two decades ago, more than 12,800 genes (46% of 27,655 protein-coding genes, Araport11, www.arabidopsis.org, accessed on March 2021) remain functionally uncharacterized. Intensive efforts have been devoted to identifying and characterizing these unknown or unannotated genes, such as *DUFs*. Several genes that contained various DUF domains have been studied in plants (e.g., *TBL/DUF231*). However, lack of genome-wide analysis of genes containing DUF domains has prevented a full understanding of their evolutionary history and biological functions.

### 3.1. Evolution, Expansion and Conservation of Plant DUF506 Genes

In the present study, examination of 23 representative species suggests that the green algae *Chlamydomonas reinhardtii* is the last common ancestor of *DUF506* prior to plant colonization of the land (Figure 1). In bryophytes (e.g., *M. polymorpha* and *P. patens*), the number of *DUF506* genes has largely increased, implying that they might have assisted with plant adaption to terrestrial conditions. Contrary to a recent report [16], the number of *DUF506* genes in different species is closely coordinated with the total number of protein-coding transcripts, regardless of genome size (Table S5). Moreover, the species that belonged to the same taxa exhibited a similar percentage of *DUF506* genes among the total genes (*DUF506*‰). For instance, between Arabidopsis and soybean, the difference of *DUF506* (0.47‰ versus 0.43‰) was significantly smaller when considering the genome size (135 Mb versus 978 Mb). Additionally, phylogenetic analysis found that in every subgroup, *DUF506* proteins from monocots or eudicots tended to cluster in the same clades, suggesting the differentiation might be subjected before lineages diverged.

In the process of evolution, genome polyploidization has occurred frequently in Eukaryotes, concomitant with gene duplication events [36]. Three types of gene duplication events have been reported, namely tandem duplication, segmental duplication, and whole-genome duplication [37]. Here, three segmental duplicated gene pairs were identified from Arabidopsis, as well as in several monocot species (Table 2 and Table S4), suggesting that the expansion of the *DUF506* gene family might have been derived from genome polyploidy events to diversify gene function. Noteworthy is that considering the chromosome positioning, phylogenetic relationship, identity of CDS and protein sequences, *At1g77145* and *At1g77160* could be an undiscovered tandem duplicated gene pair (Figure 1, Figure 2a and Figure 3; Table 1). Previous studies have demonstrated essential and multifaceted genes, such as transcription factors and protein kinases, were retained during natural selection, whereas genes with a single specialized biological function, such as transferases, had been preferentially lost [38]. *At3g25240/RXR1*, and its duplicated pair, *At3g07350*, both strongly responded to P-stress [24]. However, they showed different expression patterns when exposed to abiotic stresses or ABA treatment (Figure 6). Taken together, the present results show that *DUF506* genes from same species have experienced strong purifying positive selection after the duplication event (Ka/Ks ratio < 1, Table 2), implicating their functional redundancy under certain conditions.

Structural-modeling (secondary and tertiary structures) is increasingly considered more effective at predicting protein function than protein sequencing (primary structure) alone, because the conservation of functional foldable motifs is likely higher than the sequence [8,39]. A previous study predicted *DUF506* proteins contain a conserved (ααααβββαβαα) secondary structure pattern [20]. Comparative structural analysis found the three tandem β-sheet structures were mainly presented in motif 2 (Figure 2c), which is found in most *DUF506* proteins, whereas the α–coil structures were scattered in the neighborhood. Considering the ubiquitous presence of these secondary structures, *DUF506*s might perform conserved biological functions across species.

### 3.2. Distinct Roles of DUF506 Genes in Biological Processes

Expression profiling of Arabidopsis *DUF506* genes in different tissues, developmental stages, and under abiotic stress provided new data that could help in understanding potential biological functions. In general, transcript levels of *DUF506* genes fluctuated in various organs (Figure 5 and Figure 6a). The hypothetical tandem duplicated gene pair, *At1g77145* and *At1g77160*, displayed identical expression patterns. These results supported the hypothesis of positive selection of *DUF506* genes based on the evolutionary analysis. However, expression changes of the segmental duplicated pair diverged from that of its homolog. For example, the *At1g62420* transcript was highly abundant in roots and siliques relative to other tissues, whereas the *At1g12030* transcript was steadily expressed.

The Arabidopsis *DUF506* transcripts responded more to salt stress than to osmotic stress. As shown in Figure 6a, most Arabidopsis *DUF506* genes (10/12, except *At3g54550*) responded to salt stress, whereas only two genes (*At1g62420* and *At3g25240*) were induced by osmotic treatment. Additionally, about 75% (8/12) of the genes responded to ABA treatment, an observation that was consistent with the presence of ABRE *cis*-elements in their promoters (Figure S3). *At1g12030*, *At1g77160*, *At2g20670*, and *At2g38820*, significantly responded to both salinity and ABA treatment. *At1g62420* and *At3g25240* transcripts were strongly induced by distinct abiotic stresses (e.g., salt, osmotic, cold, and heat), but were irresponsive to ABA treatment despite the presence of ABREs in their promoter regions (Figure 5, Figure 6 and Figure S3). Overall, these results suggest that the expression of Arabidopsis *DUF506* genes are mediated through both ABA-dependent (e.g., *At1g12030* and *At1g77160*) and independent (e.g., *At1g62420* and *At3g25240*) pathways in response to environmental stimuli.

*DUF506* genes in other plant species actively responded to abiotic stress (Figures S4–S9). For example, in Brachypodium and rice, *Bradi2g58590* and *LOC_Os01g68650*, the closest homologs of *At3g25240*, were strongly up-regulated by salinity and cold stress. A similar expression pattern was observed in *At2g39650* homologs (*Bradi2g62310* and *LOC_Os01g74250*). Due to limited data availability on soybean and Medicago, the expression profiles of *DUF506* genes responding to drought stress were analyzed. *Medtr1g070970*, *Medtr2g013780*, *Medtr4g088770*, *Medtr8g093440*, and their corresponding homologs in Soybean, *Glyma.02G143100*, *Glyma.15G074800*, *Glyma.13G179200*, *Glyma.05G190100* were significantly induced by drought stress.

Nowadays, protein interaction analysis, through yeast two hybridization (Y2H) or prediction based on public inter-proteomic data, is a useful tool to provide additional insights deciphering gene functions. *At1g12030*, which is the only *DUF506* gene that clearly responds to iron starvation (Fold Change [FC] ≥ 6 in shoot, and FC ≥ 2.5 in root, GENEVESTIGATOR^®^), was predicted to interact with several iron (Fe)-deficiency response proteins (Table S6). One example is At1g47400/FEP3/IRONMAN1, which encodes a 50 amino acid long FE-UPTAKE-INDUCING PEPTIDE, predominantly expressed in the vascular tissue (e.g., phloem) of leaves and roots. Overexpression of *FEP3* significantly accumulated Fe and Mn in Arabidopsis through activation of Fe uptake genes in roots [40,41]. Additional Fe-deficiency related proteins that interactin with At1g12030, including chloroplast-localized At5g67370/CONSERVED IN THE GREEN LINEAGE AND DIATOMS 27/CGLD27, 14-3-3 protein At1g34760/ROOT HAIR SPECIFIC 5/RHS5, tonoplast-localized iron efflux transporter At5g03570/IRON-REGULATED PROTEIN 2/IREG2, and phloem-based iron transporter At1g56430/NICOTIANAMINE SYNTHASE 4/NAS4, have been demonstrated to function effectively in maintaining endogenous iron homeostasis [42,43,44,45]. Lately, a novel basic helix–loop–helix (bHLH) transcription factor, At3g19860/Upstream Regulator of IRT1/URI, was demonstrated to act as a central regulator of the iron deficient signaling pathway in Arabidopsis [46]. Chromatin immunoprecipitation followed by a sequencing (ChIP-seq) experiment revealed that URI directly bound to promoters of many iron-regulated genes, including At1g12030 and its interactors, At5g05250/IRP6, IMA1, CGLD27, and NAS4 [46]. These results suggest that At1g12030 acts in an essential URI-dependent role in responding to iron deficiency.

In addition to responding nutrient-deficiency, another Arabidopsis *DUF506* member, At2g39650, intensively interacted with four well-known salt-stress-related transcription factors, including SZF1, SZF2, ZAT10, and At3g49530/NAC062 (Table S6). Three of the ZINC finger domain-containing proteins have been thoroughly characterized and were proven to be regulators under multiple stresses (e.g., salt, drought, cold, hypoxia, and H_2_O_2_) by activating the expression of stress-inducible genes [47,48,49]. Moreover, the membrane-bound transcription factor NAC062 was reported not only to promptly activate transcriptional responses upon exposure to abiotic/biotic stress, but also to mediate the unfolded protein response (UPR) under endoplasmic reticulum (ER) stress [50,51]. Thus, considering the high induction of At2g39650 under salt stress (Figure 6a), the present results suggest its involvement in transcriptional regulation of stress-response genes by interacting with different transcription factors.

Unlike the aforementioned interactions predicted based on text mining and co-expression data, At1g77145′s interaction with at least five Calmodulin/-like proteins (CaM/CML), such as CML8, CML9, CML10, CaM6, and CaM7, was determined through protein microarrays [34]. In plants, when intracellular levels of Ca^2+^ increase, CaM/CML directly binds to Ca^2+^*via* symmetrical EF-hand motifs, then functions as an essential calcium secondary messenger by altering the activity of target proteins to contribute to signaling during developmental processes and adaptation to environmental stimuli [52]. CML8, CaM6, and CaM7 have been demonstrated to interact with the BRASSINOSTEROID-INSENSITIVE 1 (BRI1) receptor kinase [53]. The interactions inhibited the activity of BRI1, suggesting the participation of Ca^2+^ in BR signaling. Phylogenic analysis indicated that CML8, CML9, and CML10 belong to the same CML subgroup in Arabidopsis, and actively responded to various environmental stimuli [54]. The *CML8* transcript has been reported to be induced by salicylic acid (SA) and salt treatment [55]. *CML9* mutants exhibited hypersensitivity to ABA, and elevated salt and drought tolerance through up-regulation of stress-response genes, suggesting that AtCML9 is involved in the ABA-dependent pathway responding to abiotic stress [56]. Differently, through interacting with the Ascorbic acid (AsA) biosynthesis-related enzyme phosphomannomutase (PMM), CML10 augmented the activity of PMM and consequently modulated oxidative stress responses [57]. Moreover, CML8 and CML9 have been reported to play roles in plant immune response [58,59]. Searching the Calmodulin Target Database [60], a classic 1-12 motif ([FILVW]xxxxxxxxxx[FILVW], two bulky hydrophobic residues spaced by 10 amino acids residues) was identified in motif 1 of At1g77145. Further analysis showed different types of CaM binding motifs were scattered in twelve of the Arabidopsis *DUF506* proteins (Table S7). Presumably, At1g77145, and perhaps other *DUF506* members, might specifically bind to certain CaMs/CMLs in the presence of Ca^2+^, leading to a structural conformation change or a shift in enzymatic activity, and ultimately influencing downstream stress-response gene expression.

The silique-specific At3g54550 potentially interacts with At2g38820 (Table S6), which is constitutively expressed in vivo, implying a possible chimeric complex consisting of different *DUF506* proteins, might perform regulatory functions during the reproductive stage, especially during seed development.

Overall, the broad response of *DUF506* genes to various abiotic stresses and the predicted interaction with various functional proteins suggest that Arabidopsis *DUF506* proteins have a diverse range of regulatory functions in response to abiotic stress (e.g., At2g39650 and At1g77145) or nutrient deficiency (e.g., At1g12030 and At3g25240).

## 4. Materials and Methods

### 4.1. Identification and Phylogenetic Analysis of DUF506 Protein in Plants

In this study, 211 *DUF506* candidate genes (Pfam accession: PF04720, ID: PDDEXK_6, Description: PDDEXK-like family of unknown function, https://pfam.xfam.org/) were identified from 17 plant species. The protein sequences, cDNA sequences, DNA sequences, upstream 2 kb genomic DNA sequences, and protein-coding sequences (CDS) of *DUF506* were downloaded from Phytozome v12.1.6 (*Amborella trichopoda* v1.0, *Arabidopsis thaliana* Araport11, *Capsella rubella* v1.0, *Brassica rapa* FPsc v1.3, *Cucumis sativus* v1.0, *Glycine max* Wm82.a2.v1, *Phaseolus vulgaris* v2.1, *Medicago truncatula* Mt4.0 v1, *Ricinus communis* v0.1, *Vitis vinifera* Genoscope.12X, *Daucus carota* v2.0, *Solanum lycopersicum* iTAG2.4, *Oryza sativa* v7_JGI, *Brachypodium distachyon* v3.1, *Hordeum vulgare* r1, *Sorghum bicolor* v3.1.1, and *Zea mays* PH207 v1.1). In genes undergoing alternative splicing, the longest transcript was used for downstream analysis. Predicted partial proteins carrying the signature domain were filtered out. The theoretical isoelectric point (pI) of *DUF506* proteins were calculated by Sequence Manipulation Suite (https://www.bioinformatics.org/sms2/, Sequence analysis tool—Protein Isoelectric Point) [61]. The subcellular localization was predicted by using WoLF PSORT program (https://wolfpsort.hgc.jp/).

The amino acid sequence alignment of *DUF506* genes was performed by Clustal Omega1.2.2 (https://www.ebi.ac.uk/Tools/msa/clustalo/). The alignment was used to construct an unrooted phylogenic tree using the neighbor-joining method with 1000 bootstrap replications using Fast Tree. Both programs were carried out within Geneious Prime^®^ software (v.2020.2.3.).

### 4.2. Gene Structure and Conserved Motif Analysis

The exon-intron distributions of *DUF506* genes were graphically generated by Gene Structure Display Server 2.0 [62] (http://gsds.gao-lab.org/) using coding sequences and their corresponding genomic DNA sequences under default settings. The conserved motifs of *DUF506* protein were predicted by using the MEME web server [27] (v5.1.1, http://meme-suite.org/tools/meme) with the maximum number of motifs set at 10, and optimum width of motifs between 5 and 100 amino acids. The secondary structure of *DUF506* protein was predicted by using the JPred4 web server [63] (A Protein Secondary Structure Prediction Server, http://www.compbio.dundee.ac.uk/jpred4/index_up.html).

### 4.3. Chromosomal Localization, Gene Duplication and Syntenic Analysis

Chromosomal locations of Arabidopsis *DUF506* proteins were visualized by using ePlant web tool (https://bar.utoronto.ca/eplant/). Gene duplication events were analyzed by using the Multiple Collinearity Scan toolkit [64]. The precalculated nonsynonymous (Ka) and synonymous (Ks) substitutions of each duplicated Arabidopsis *DUF506* genes were obtained from Plant Genome Duplication Database (PGDD, http://chibba.agtec.uga.edu/duplication/).

### 4.4. Subcellular Localization of Arabidopsis DUF506 Protein

The coding regions, except for the terminator codon of selected Arabidopsis *DUF506* genes, were amplified and then ligated into the destination vector, pEarleyGate 103 [29], to generate a AtDUF506-GFP fusion construct under the control of the CaMV35S promoter. Constructs were transformed into Arabidopsis (Col-0) mesophyll protoplast as described previously [30]. Twelve hours after transformation, fluorescence was detected using a Leica TCS SP8 confocal laser-scanning microscope. All experiments were repeated at least three times and representative images are displayed in figures.

### 4.5. Stress-Related Cis-Elements Analysis

The PlantCARE program [65] (http://bioinformatics.psb.ugent.be/webtools/plantcare/html/) was employed to analyze the 2 kb upstream genomic sequences of plant *DUF506* genes.

### 4.6. Expression Analysis of Arabidopsis DUF506 Genes via qRT-PCR

*Arabidopsis thaliana* (ecotype Col-0) seeds were sterilized in 70% ethanol and 20% bleach as described previously [66]. After stratification for 3 days at 4 °C, sterilized seeds were directly sown in half strength (1/2) Murashige and Skoog (MS) medium (Caisson Labs) supplemented with 1% sucrose (pH 5.7) and 0.05% MES (*w*/*v*). Seeds were germinated and grown in a growth chamber maintained at 22°C (16-h-light and 8-h-dark cycle, 120 µmol^−2^s^−1^ light intensity). The roots and shoots of 10-day–old seedlings, and the roots, stems, rosette leaves, flowers and siliques of mature Arabidopsis plants were used for tissue-specific expression analysis. For salt stress, osmotic stress, or ABA treatment, 7-day-old Arabidopsis seedling were transferred into 1/2 MS liquid medium supplemented with 150 mM NaCl, 300 mM mannitol or 100 µM (±)-*cis, trans*-ABA (Sigma), and the stress treatments were applied for 24 hr. A fresh medium-only control was conducted in parallel. Samples were rinsed with demineralized water, shoot and root tissues were harvested separately, snap-frozen in liquid N_2_ and stored at −80 °C until further use.

The qRT-PCR experiments were performed according to previous studies [66]. All the experiments were repeated at least three times using cDNA prepared from two biological replicates. Primers used in the study are listed in Supplemental Table S8.

### 4.7. Protein-Protein Interaction Analysis

STRING-DB [33] (v11.0, https://string-db.org/), AtPIN [67] (Arabidopsis thaliana protein interaction network, https://atpin.bioinfoguy.net/cgi-bin/atpin.pl), and AI-1 [68] (Arabidopsis Interactome-1, http://interactome.dfci.harvard.edu/A_thaliana/index.php) online search tools were used to predict the putative protein-protein interaction networks with candidate Arabidopsis *DUF506* proteins using default settings.

## 5. Conclusions

In this study, 211 *DUF506* genes in 17 land species were identified and were conducted to estimate evolutionary analysis. Gene structure and conserve motif analysis indicated the conservation and dispersal of *DUF506* during evolution. Furthermore, expression profiling and protein interaction predictions suggest that Arabidopsis *DUF506* genes might be involved in plant resilience to environmental stresses.

## Figures and Tables

**Table 1:** Physicochemical properties of *DUF506* family members in Arabidopsis.

GeneID	# of A.A	p.I.	Subcellular Localization	Annotation (Phytozome v12.0)
At1g12030	296	5.85	cyto	phosphoenolpyruvate carboxylase
At1g62420	284	7.46	chlo; cyto	*DUF506* family protein
At1g77145	261	8.96	nucl	transmembrane protein
At1g77160	264	8.26	nucl	hypothetical protein
At2g20670	295	6.37	nucl	sugar phosphate exchanger
At2g38820	311	8.53	nucl	DNA-directed RNA polymerase subunit beta-beta protein
At2g39650	292	6.79	nucl	cruciferin (*DUF506*)
At3g07350	299	5.47	nucl	sulfate/thiosulfate import ATP-binding protein
At3g22970	371	5.02	extr	hypothetical protein
At3g25240	282	7.61	nucl	sulfate/thiosulfate import ATP-binding protein
At3g54550	289	8.63	chlo	DNA-directed RNA polymerase subunit beta-beta protein
At4g14620	342	8.28	cyto; cytoplas	hypothetical protein
At4g32480	288	7.66	nucl	sugar phosphate exchanger

**Table 2:** Synteny analysis of *DUF506* genes in Arabidopsis.

Duplicated Gene Pairs	Ka	Ks	Ka/Ks	Duplication Type	Type of Selection
*At1g12030/At1g62420*	0.28	1.03	0.2718	Segmental	Purify selection
*At3g07350/At3g25240*	0.46	1.76	0.2614	Segmental	Purify selection
*At3g22970/At4g14620*	0.21	1.05	0.2000	Segmental	Purify selection

## Data Availability

The data and materials that support the findings of this study are available from the corresponding author upon reasonable request.

## Supplementary Materials

The following are available online at https://www.mdpi.com/article/10.3390/ijms222111442/s1.
