# Supplementary material for: Genome-Wide Identification and Transcriptional Analysis of Arabidopsis DUF506 Gene Family
Source: Int J Mol Sci. 2021 Oct 23;22(21):11442. doi: 10.3390/ijms222111442 (PMC8583954; doi:10.3390/ijms222111442)
Supplement: Supplementary file 1 [file ijms-22-11442-s001.zip › Supplemental Figures.pdf]

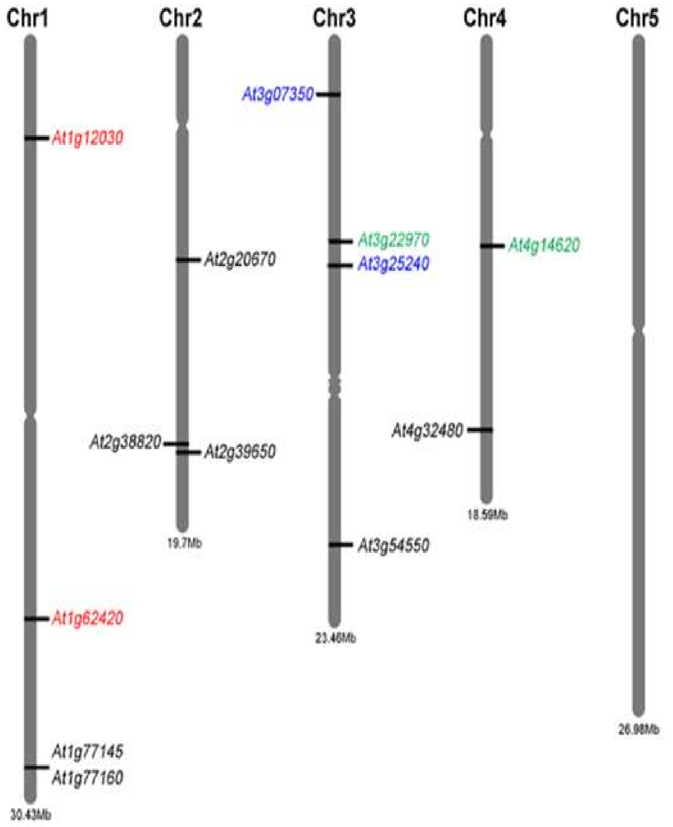

Figure S1. Chromosomal position of the 13 *Arabidopsis DUF506* genes. The graph was generated from available data on the TAIR database and visualized using the ePlant viewer (https://bar.utoronto.ca/eplant/). The segmental duplicated gene pairs are indicated in same colors.

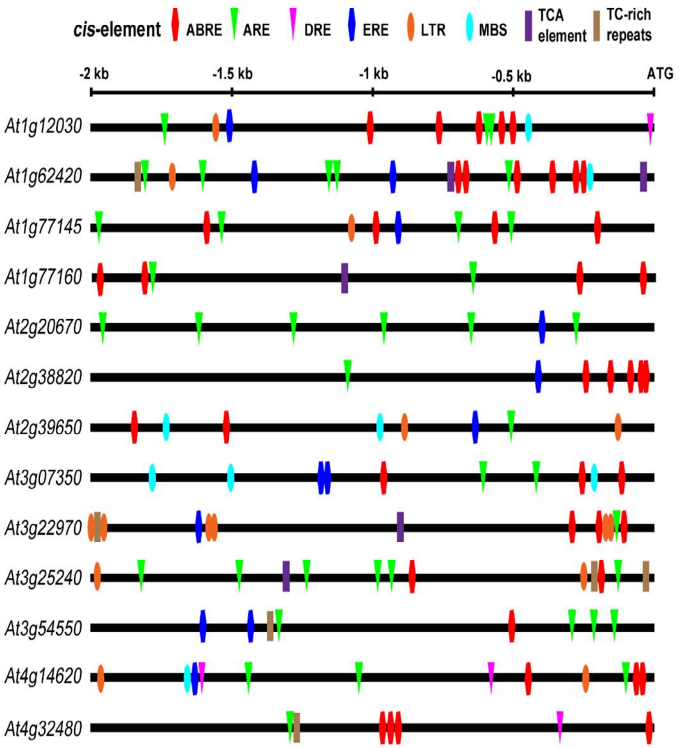

Figure S3. The cis-elements in *Arabidopsis DUF506* promotor regions. Eight different cis-elements were identified using PlantCARE in 2 kb upstream region from start codon, related to hormone and stress response. ABRE, cis-element involved in abscisic acid responsiveness; ERE, ethylene responsive element; TCA-element, cis-element involved in salicylic acid responsiveness; ARE, cis-element essential for anaerobic induction; DRE, cis-element related to dehydration; LTR, cis-element related to low temperature; MBS, MYB-binding site involved in drought inducibility; TC-rich repeats, cis-element involved in defense and stress response.

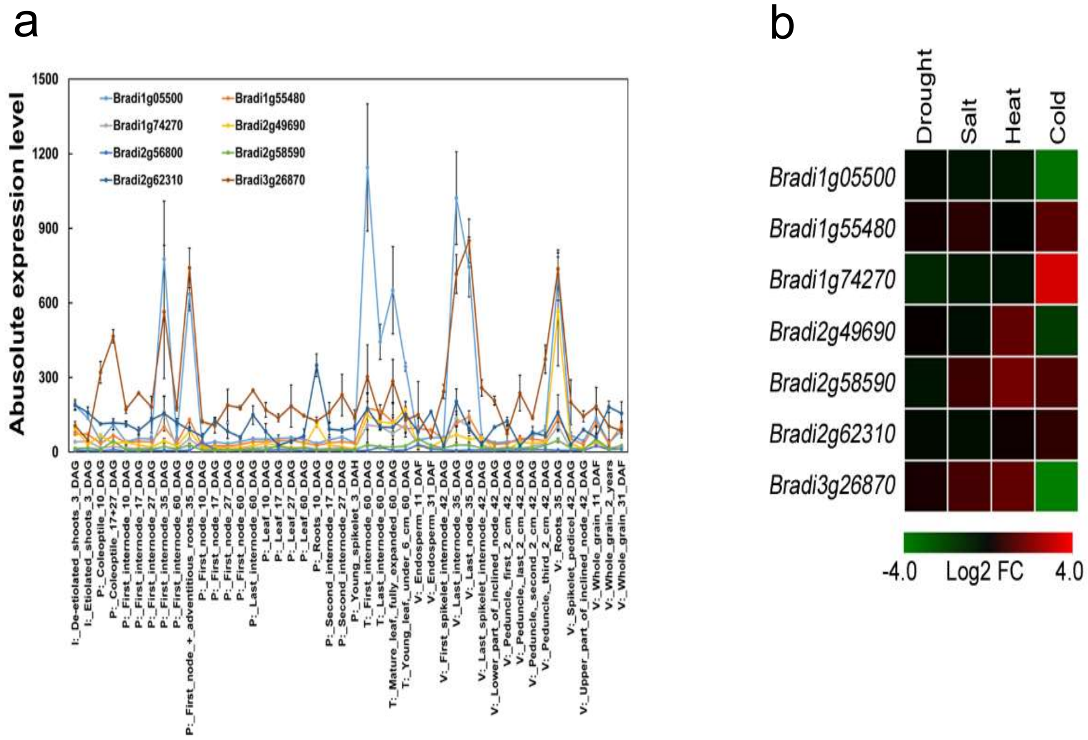

Figure S4. Expression profiles of *Brachypodium DUF506* genes in different tissues (a) and responding to abiotic stress (b). The green-black-red tricolor bar beside the heat map represents their relative fold changes.

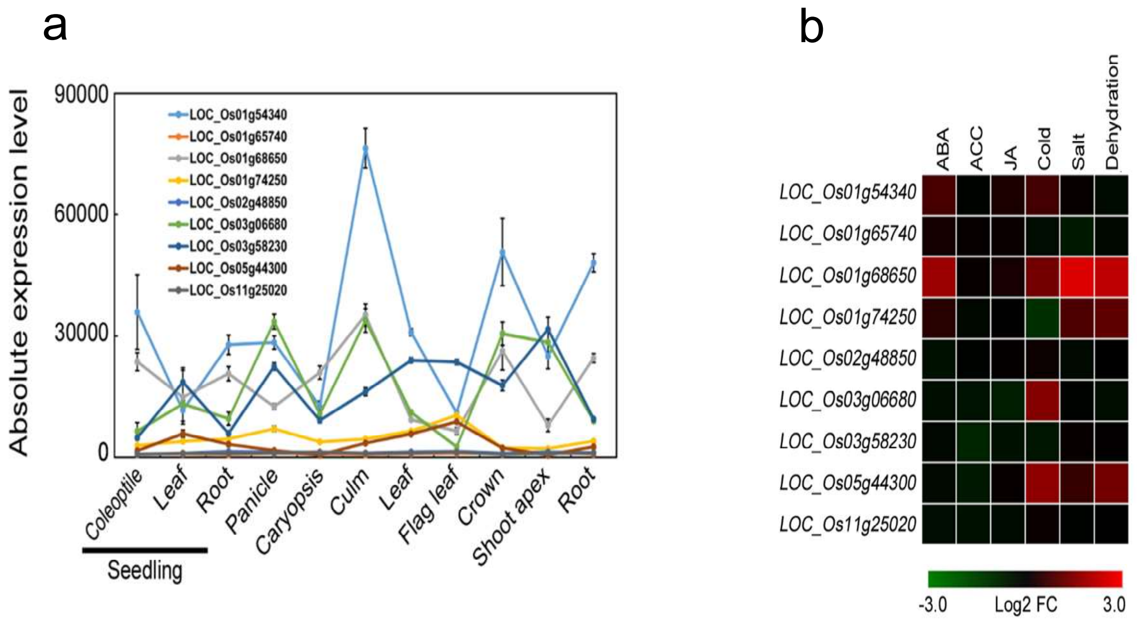

Figure S5. Expression profiles of rice *DUF506* genes in different tissues (a) and responding to abiotic stress or hormone treatment (b). The green-black-red tricolor bar beside the heat map represents their relative fold changes.

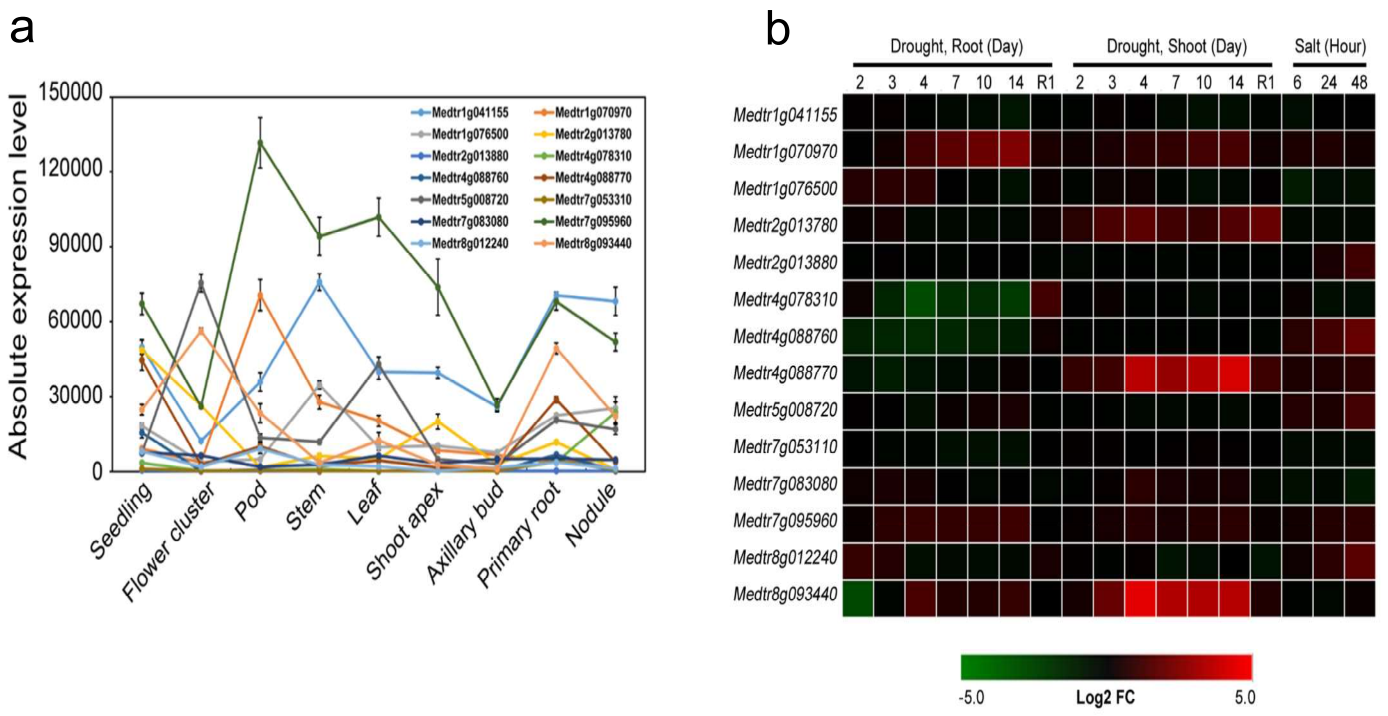

Figure S6. Expression profiles of *Medicago DUF506* genes in different tissues (a) and responding to abiotic stress (b). The green-black-red tricolor bar beside the heat map represents their relative fold changes.

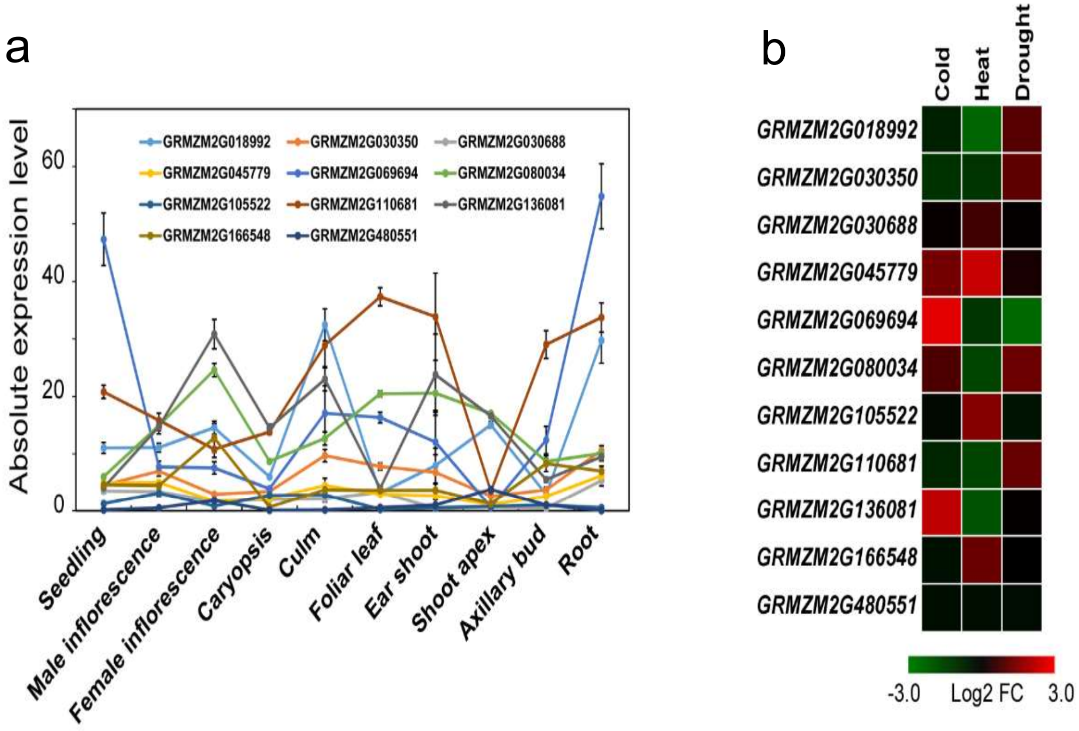

Figure S7. Expression profiles of maize *DUF506* genes in different tissues (a) and responding to abiotic stress (b). The green-black-red tricolor bar beside the heat map represents their relative fold changes.

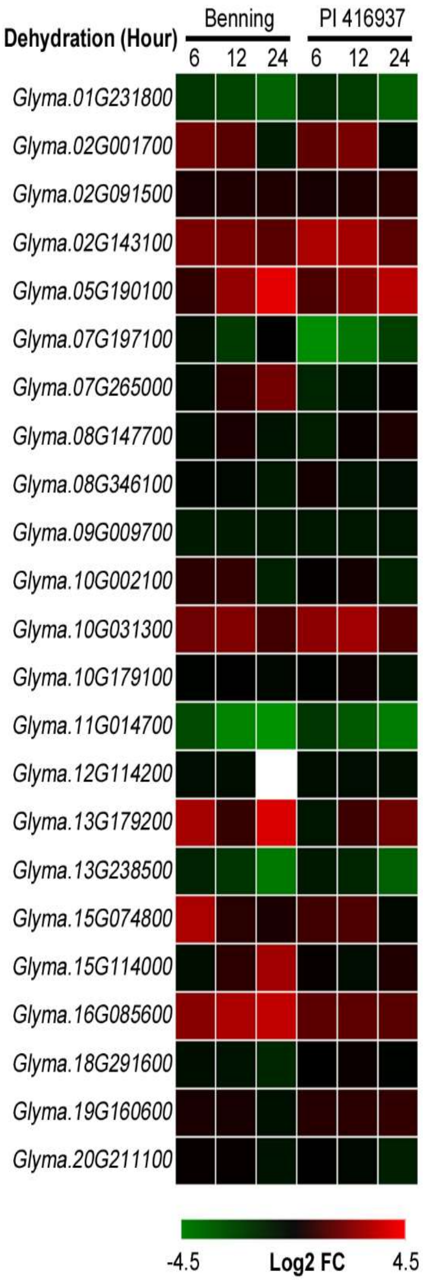

Figure S8. Expression profiles of soybean *DUF506* genes under dehydration stress in drought-sensitive (Benning) genotype and drought-tolerant (PI 416937) genotype, respectively. The green-black-red tricolor bar beside the heat map represents their relative fold changes.

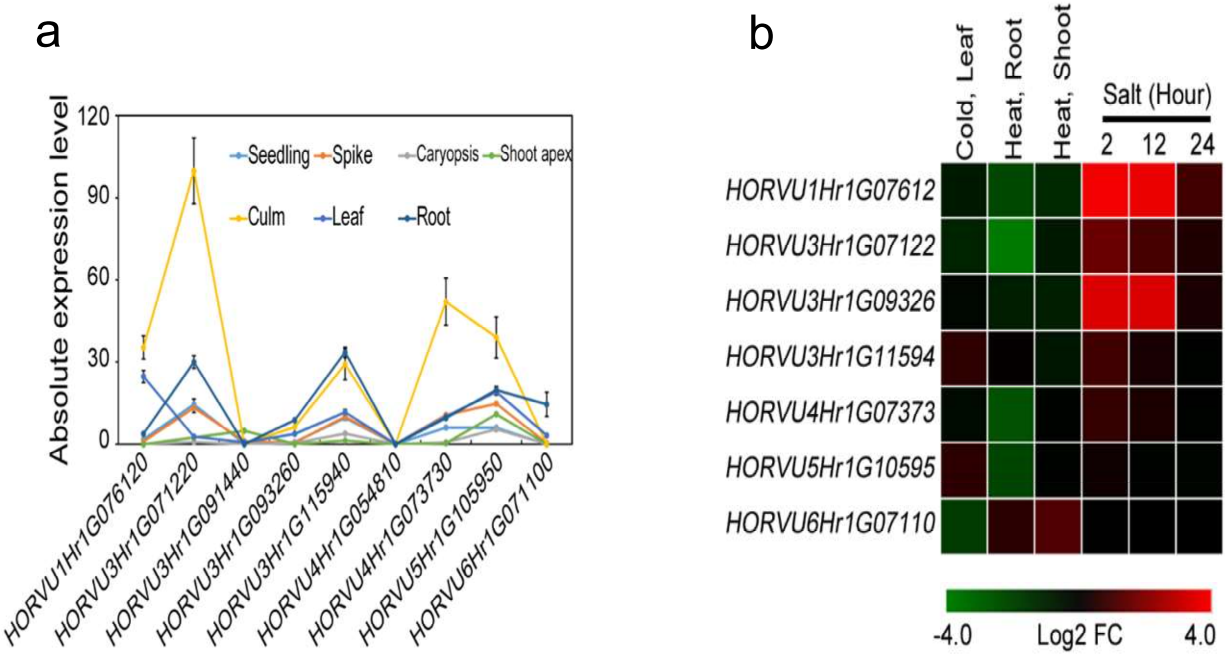

Figure S9. Expression profiles of barley *DUF506* genes in different tissues (a) and responding to abiotic stress (b). The green-black-red tricolor bar beside the heat map represents their relative fold changes.
